# Distal Humeral Fixation of an Intramedullary Nail Periprosthetic Fracture

**DOI:** 10.1155/2013/690906

**Published:** 2013-04-10

**Authors:** Hiren M. Divecha, Hans A. J. Marynissen

**Affiliations:** Department of Trauma & Orthopaedic Surgery, East Lancashire Hospitals NHS Trust, Haslingden Road, Blackburn BB2 3HH, UK

## Abstract

Distal humeral periprosthetic fractures below intramedullary nail devices are complex and challenging to treat, in particular due to the osteopenic/porotic nature of bone found in these patients. Fixation is often difficult to satisfactorily achieve around the intramedullary device, whilst minimising soft tissue disruption. Descriptions of such cases in the current literature are very rare. We present the case of a midshaft humeral fracture treated with a locking compression plate that developed a nonunion, in a 60-year old female. This went on to successful union after exchange for an intramedullary humeral nail. Unfortunately, the patient developed a distal 1/5th humeral periprosthetic fracture, which was then successfully addressed with a single-contoured, extra-articular, distal humeral locking compression plate (Synthes) with unicortical locking screws and cerclage cables proximally around the distal nail tip region. An excellent postoperative range of motion was achieved.

## 1. Introduction

Fractures occurring below a humeral intramedullary nail are rare and present a unique challenge to management. Distal fixation is generally difficult due to osteopenia/porosis, and the presence of an intramedullary nail also poses difficulty in achieving reasonable proximal fixation. We present a case of a periprosthetic distal humeral fracture (occurring below an intramedullary humeral nail inserted for revision fixation of a midshaft humeral nonunion) that was successfully managed using a contoured locking plate. 

## 2. Case History

**Figure 1:**
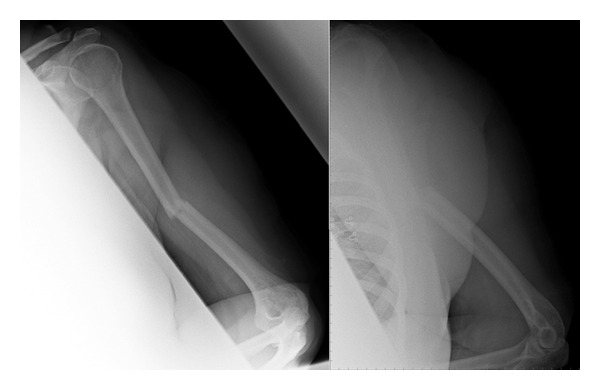
AP and lateral radiographs of midshaft humeral fracture.

A 60-year-old right hand dominant occupational therapist presented to the emergency department with a left arm injury following a mechanical fall down a set of stairs. Her past medical history included controlled hypertension and mild asthma. She had a BMI of 36, was a nonsmoker, and consumed approximately 8–10 units of alcohol per week. A transverse, angulated, midshaft fracture of the left humerus (Figure 1) was confirmed, and the patient underwent an uneventful open reduction and internal fixation with a locking compression plate (Synthes). At 10 weeks postoperatively, the patient complained of arm pain, and plain radiographs showed no obvious fracture healing. Humeral bracing and three weeks of low intensity ultrasound treatment (Exogen, Smith, and Nephew) were used.

**Figure 2:**
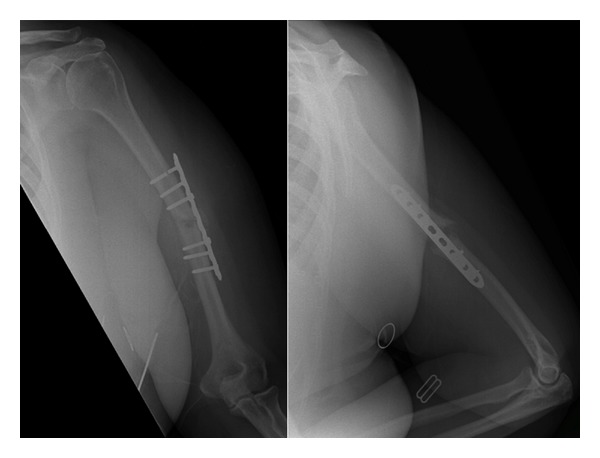
AP and lateral radiographs demonstrating proximal screw breakage and oligotrophic nonunion.

At five months postoperative, radiographic review unfortunately confirmed an oligotrophic non-union with proximal screw breakage (Figure 2). This was addressed with removal of metalwork, non-union debridement, and insertion of a locked, reamed, 8.5 mm intramedullary humeral nail (Synthes). 

**Figure 3:**
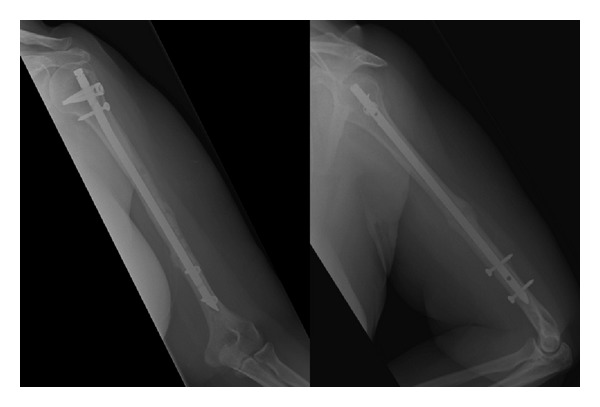
AP and lateral radiographs showing fracture union following revision to intramedullary nail.

Radiographs at 2 months following this revision demonstrated successful midshaft fracture union (Figure 3); however the patient complained of pain at the elbow with movement. This was felt to be due to the distal locking screws, which were therefore removed at 3 months following revision.

**Figure 4:**
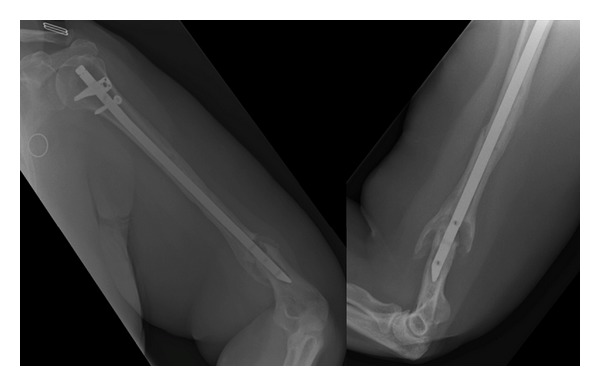
AP and lateral radiographs demonstrating hypertrophic nonunion of distal humeral periprosthetic fracture.

The elbow pain persisted despite removal of the distal locking screws and appropriate physiotherapy. There was no history of trauma. In particular, she experienced pain in the terminal 30° of extension. Plain radiographs demonstrated a transverse fracture of the distal 1/5th of the humerus propagating through the distal locking screw site, which, despite conservative treatment with bracing for 2 months, progressed to a hypertrophic non-union (Figure 4). 

**Figure 5:**
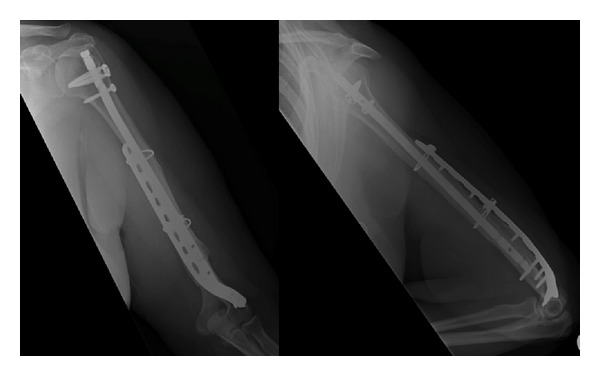
AP and lateral radiographs demonstrating union of distal humeral periprosthetic fracture.

**Figure 6:**
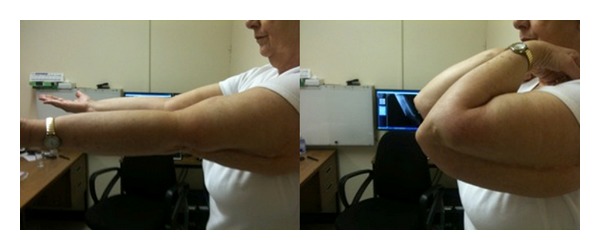
Photographs demonstrating elbow ROM.

This non-union was addressed via a posterior triceps split, debridement of the non-union site, application of DBX putty (Synthes), and fixation with an extra-articular distal humeral locking compression plate (Synthes). Proximal fixation was achieved with two unicortical locking screws supplemented with two tensioned 1.0 mm cables. This was uneventful, and progressive postoperative physiotherapy was initiated. At 5 months following this fixation, symptomatic and radiographic union was confirmed (Figure 5) with a full range of movement (Figure 6).

## 3. Discussion

Periprosthetic distal humeral fractures are uncommon and represent complex injuries to manage. There have been a number of reports in the literature on the management of distal humeral fractures occurring near the humeral component of either a shoulder arthroplasty or an elbow arthroplasty. Fewer still are descriptions of such fractures occurring in the distal humerus below an intramedullary nail. The region distal to the locking screws of an intramedullary humeral nail represents an area of increased stress resulting in an increased risk of fracture. Risk factors associated with periprosthetic distal humeral fractures include advancing age, female sex, osteoporosis, and rheumatoid arthritis [1]. We are aware of two cases reports of distal humeral periprosthetic fractures following intramedullary humeral nail fracture fixation. None have been reported following the use of an intramedullary humeral nail for revision fixation of a midshaft humeral non-union. 

Shin et al. [2] describe the case of a young patient (26 years old), without suggestion of osteoporosis, who received a proximal intramedullary humeral nail for treatment of a proximal humeral fracture. The periprosthetic fracture occurred 4 months postoperatively and was a long spiral distal third fracture below the nail. This was then managed successfully by removal of the nail and then conservative management with splints and functional bracing [2]. We are uncertain if removal of the original nail was required in this case given that the fracture was well distal to the nail. 

Sarraf et al. describe a distal humeral fracture in a 72-year-old man following intramedullary fixation of a midshaft humeral fracture [3]. Fracture union was successfully achieved with bicondylar locking plate fixation using a “miss-a-nail” technique, supplemented with cerclage wires. This was performed through a posterior approach with an olecranon osteotomy. Whilst the outcome of this reported case was successful, we suggest that stable fixation with a single-contoured locking plate (supplemented proximally with cerclage wires if needed) is achievable without the need for bicondylar plating and the associated extensive soft tissue damage.

Certainly, the development of locking plate technology has changed the way these and other complex periprosthetic fractures are managed, especially in osteoporotic bone [4]. Greater fracture stability can be achieved than with conventional plates, and the overall construct rigidity can be controlled by altering the plate length and screw: hole ratio [5]. Periosteal stripping/damage is minimized as these contoured locking plates are designed to be placed submuscularly/epiperiosteally, thereby maximizing fracture healing potential.

## 4. Conclusion

Whilst periprosthetic fractures of the distal humerus are uncommon, they present a unique challenge to management given the osteopenic/porotic nature of the bone. Our presented case highlights the importance of vigilant followup. More importantly, we demonstrate how these difficult fractures can be managed successfully with the use of a single-contoured locking plate with a long working length to prevent excessive rigidity and reduce strain on the proximal fixation provided by a combination of unicortical locking screws and cables around the region overlapped with the humeral nail. Soft tissue management is paramount to maintaining vascularity to the fracture region and maximising healing potential.
